# Exosomal miR-105-5p derived from bladder cancer stem cells targets for GPR12 to promote the malignancy of bladder cancer

**DOI:** 10.1186/s12894-023-01326-2

**Published:** 2023-10-03

**Authors:** Gaojian Pan, Bo Jiang, Zhongquan Yi, Jiuhu Yin, Yadong Liu

**Affiliations:** 1 Department of Urology, The Affiliated Yancheng Hospital Of Southeast University Medical School, NO. 2 Xindu West Road, Yancheng, 224001 China; 2 Department of Urology, The Yancheng School of Clinical Medicine of Nanjing Medical University, NO. 2 Xindu West Road, Yancheng, 224001 China; 3 Department of Urology, Affiliated Hospital 6 of Nantong University, NO. 2 Xindu West Road, Yancheng, 224001 China

**Keywords:** Cancer stem cell, Bladder cancer, Exosome, miR-105-5p, GPR12

## Abstract

Bladder cancer stem cells (BCSCs) are considered as the root cause of BC initiation and recurrence, and exosomes derived from BCSCs (CSCs-exo) are the vital tool for establishing a stable tumor microenvironment. miR-105-5p has been revealed to promote tumor growth in a variety of cancers, but the effects on BC are still not included.Characteristics of CSCs-exo were examined by transmission electron microscope and nanoparticle tracking analysis. PKH67 dye was used to observe the cellular uptake of exosomes. Cell viability, migration and invasion were detected by CCK-8, wound healing and transwell invasion assays, respectively. The interaction between miR-105-5p and GPR12 was verified by luciferase activity assay. Xenografts were induced in the nude mice, and H&E staining method was applied to analyze the histological changes of xenografts. CSCs-exo efficiently promoted BC cell viability, migration and invasion. miR-105-5p was highly expressed in CSCs and CSCs-exo treatment significantly upregulated the expression of miR-105-5p in BC cells.GPR12 was subsequently verified to be the target gene of miR-105-5p, and overexpression of GPR12 abrogated the effects of miR-105-5p on BC cell growth and metastasis. Reversely, the anti-tumor function of miR-105-5p antagomir was observed in the xenograft mice.CSCs aggravated the malignancy of BC partly through transmitting exosomal miR-105-5p to BC cells to inhibit the expression of GPR12, which developed a novel aspect for CSC-targeted therapies.

**Supplementary Information:**

The online version contains supplementary material available at 10.1186/s12894-023-01326-2.

## Introduction

Bladder cancer (BC) is one of the most common malignant tumors in urinary system, and 90% of BC usually originates from urothelium [1]. At present, cystoscopy and exfoliative cytology are the main diagnostic approaches of BC, however, sensitivities of these approaches are limited [2]. Liquid biopsy is a noninvasive technology based on human body fluid that is applied to obtain relevant disease information, and exosomes are one of the primary detection objects [3]. For treatments of BC, surgical resection is the first choice for BC patients, but some patients may relapse and progress to muscle-invasive and metastatic BC. These patients are prone to suffer from chemotherapy resistance after systemic chemotherapy, eventually leading to poor prognosis [4]. According to the global cancer statistics in 2018, there were approximately 549,393 new cases of BC and 199,922 deaths [5]. The exact pathogenic mechanism of BC has not yet been fully elucidated. In the past 30 years, the researches of BC have made only little progress due to the lag of exploring molecular biological characteristics of BC [6]. Therefore, in-depth studies of the pathogenesis of BC at the molecular level can provide novel ideas for diagnosis and treatment of BC.

Cancer stem-like cells (CSCs) are a subgroup of tumor cells. They possess powerful stem cell-like properties, including self-renewal, drug resistance, and tumor initiation [7]. Interaction between CSCs and the circumjacent microenvironment has been demonstrated to be the key to tumor progression. Stemness and functions of CSCs greatly depend on the signal stimulation of the microenvironment, which helps to promote tumor survival and development [8]. Overwhelming evidence shows that exosomes are crucial intercellular transmitters of information in the microenvironment of CSCs [5]. Suppression of information transmission will damage the self-renewal characteristic of CSCs, thereby inhibiting tumor growth, but the involved cellular and molecular mechanisms are not elucidated. Hence, revealing the molecular mechanism of bladder cancer stem cell-like cells (BCSCs)-mediated signaling is of great significance.

Exosomes are extracellular vesicles with a diameter of 50–150 nm that exist in the body fluids of many eukaryotes, TSG101, CD9 and CD63 are the typical surface markers of exosomes [8]. In recent years, studies have demonstrated that exosomes contain various proteins, nucleic acids and lipids. Among them, microRNAs (miRNAs) are small nucleotides with about 22–25 nucleotides, participate in regulatory processes of almost all types of cancers. They can be packaged in exosomes and transmitted to target cells, thereby influencing functions of the cells [9]. Furthermore, the phospholipid bilayer structure of exosomes could protect the internal miRNAs from being degraded. The intercellular communication mediated by miRNAs greatly contributes to the signal transmission between cancer cells [10]. CSCs can mediate tumor cells through exosomal miRNAs to establish a local and remote microenvironment that is suitable for tumor growth [11]. miR-105-5p plays an oncogenic role in a variety of tumors, such as non-small cell cancer [12] and triple-negative breast cancer [13], however, the function of miR-105-5p is still not clear in BC. Therefore, investigating the role of exosomal miR-105-5p derived from CSCs is crucial to tumor diagnosis and treatment.

## Materials and methods

### Cell culture

Human BC cell lines T24 (BNCC100121) and EJ (BNCC342285) were purchased from Bena Culture Collection and cultured in 90% RPMI-1640 (31,800,022; Gibco) + 10% FBS (Gibco) at 37 °C in an atmosphere condition of 5% CO_2_. After 80% confluence, the cells were used for subculture.

### Cell transfection

T24 and EJ cells were co-cultured with CSCs-exo (10 μl) for 48 h or transfected with miR-105-5p mimic (40 nM), miR-105-5p inhibitor (80 nM), pcDNA3.1/GPR12 (15 nM), or their negative controls (designed and synthesized by Shanghai IBS Biotech Co., Ltd.) using Lipofectamine® 2000 (Invitrogen) for 48 h, and the functional experiments were subsequently carried out.

For the preparation of in vivo experiments, miR-105-5p antagomir or NC antagomir (20 μM; Shanghai IBS Biotech Co., Ltd.) was transfected into CSCs derived from T24 cells, and CSCs-exo was isolated from the cells until antagomir miR-105-5p was stably expressed.

### Sphere formation and identification

Until cell confluence reached 90%, T24 cells were digested using trypsin–EDTA solution (R001100; Gibco). The cells were resuspended in serum-free DMEM/F12 supplemented with 20 μl/ml B27, 20 ng/ml of epidermal growth factor, and 20 μg/ml insulin (all from Gibco) and inoculated in the ultra-low adhesion culture dish (3261; Corning) at the density of 2 × 10^3^ cells/ml. After culturing for seven days, spheres were subjected to immunofluorescence assay according to the previous study [14] for validating the CSC markers, including Nestin and CD105.

The tumor spheres were disassociated into single cells and miR-105-5p inhibitors or its control were transfected into the cells. The cells were collected and cultured to propagate the next generation of spheres.

### Western blotting assay

Proteins of exosomes and tumor cells were extracted using Pierce™ IP lysis buffer (87,787; Thermal Fisher). 40 μg of the protein was resolved in each well of 10% SDS-PAGE (P0690; Beyotime) and transferred to PVDF membranes (FFP24; Beyotime). Then, the blots were blocked by 5% fat free milk and incubated with anti-CD63 (1:1000; MAB50482-100; R&D System), anti-TSG101 (1:1000; ab125011; Abcam), anti-CD9 (1:1000; ab236630; Abcam), anti-calnexin (1: 20,000; ab92573; Abcam), anti-GPR12 (1:3000; PA5-33,617; Invitrogen) and anti-GAPDH (1:5000; ab8245; Abcam) primary antibodies at 4 °C overnight. The day after, rabbit anti mouse IgG secondary antibody (1:2000; ab6728; Abcam) was incubated with the membranes at room temperature for 2 h. GAPDH was used as control. Finally, protein bands were visualized using the Ultra High Sensitivity ECL Substrate Kit (ab133409; Abcam).

### Isolation of exosome

Tumor spheres cultured for 14–21 days were collected at about 1 × 10^6^ cells/ml. The cell suspension was filtered by a 0.22‐μm filter (Millipore) to remove cell debris, followed by ultra-centrifuged at 100,000 g for 60 min to obtain the exosome pallets. Next, the pellets were rinsed and resuspended in PBS for further use.

### Transmission electron microscope (TEM) assay

Exosomes were added onto the Formvar-carbon copper grids, absorbed and then fixed with 2% paraformaldehyde (C104188; Aladdin) under room temperature. Next, 50 μl of 3% phosphotungstic acid solution (pH 7; P100467; Aladdin) was used for negative staining for 10 min. The excessive solution was wiped off and the exosomes were observed under a TEM (H-9500; Hitachi Ltd.) after the copper grids were dried.

### Nanoparticle tracking analysis (NTA)

Exosomes were resuspended in PBS and subjected to NanoSight NS300 (Malvern Panalytical Ltd.) to analyze the particle sizes.

### PKH67 staining assay

Exosome uptake of EJ and T24 cells was analyzed by PKH67 staining assay using the kit according to the manual (MX4023-100UL; Shanghai Maokang Biotech Co., Ltd.). Briefly, 2** × **10^7^ exosomes were collected and resuspended in 1 ml Diluent C. 4 μl of PKH67 solution was added into the resuspension and incubated for 5 min. Then, the PKH67-labeled exosomes were incubated with EJ and T24 cells for 24 h and the cellular location of the exosomes was observed by a fluorescence microscope. DAPI was used to stain the nuclei of the tumor cells.

### Bioinformatics prediction

Putative targets of miR-105-5p were predicted by miRDB (http://mirdb.org/), miRWalk (http://mirwalk.umm.uni-heidelberg.de/) and TargetScan (http://www.targetscan.org/vert_71/). The preliminary results were filtered following the criteria: target score > 80 (miRDB), bindingp = 1 (miRWalk), cumulative weighted context +  + score ≤ -0.1 (TargetScan). 20 genes were contained in the intersection part among the results of three databases, as analyzed by Venny 2.1.0 (https://bioinfogp.cnb.csic.es/tools/venny/index.html).

### Cell counting kit 8 (CCK-8)

Cell viability was detected by CCK-8 assay (HY-K0301; MedChemExpress Co., Ltd.). In brief, 1 × 10^5^ EJ or T24 cells were collected and plated into a 96-well plate and after transfection or incubation with the engineered CSCs-exo. 10 μl of CCK-8 reagent was added to each well of the plate and incubated at 37 °C. The optical densities were evaluated at 0 h, 24 h, and 48 h after incubation.

### Wound healing assay

EJ and T24 cells were inoculated into a 6‑well plate and cultured at 37 °C in 5% CO_2_ until cell confluence reached 90%. Then, a line was scraped into the monolayer cell using the tip of a micropipette. PBS was used to rinse the plate twice to remove the non-adherent cells and cultured in a serum‑free DMEM for 24 h. Images were observed and photographed at 0 h and 24 h under a microscope, and the wound healing rates were analyzed using Image J software (version 1.8.0).

### Transwell invasion assay

EJ and T24 cells were resuspended at the density of 2 × 10^5^ cells/ml in serum-free RPMI-1640 medium and starved for 24 h. Transwell chambers (3422; Corning) coated with Matrigel® (Corning) were inserted in a 24-well plate. Cell suspension was added into the upper Transwell chambers, and 600 μl of RPMI-1640 with 20% FBS was added into the lower chambers. After incubated for 24 h, the cells remained in the upper chambers were gently removed, and cells invaded to the lower chambers were fixed with methanol and stained with 0.1% crystal violet. The stained cells were observed and counted under a light microscope (XSP-1200A; Shanghai CSOIF Co., Ltd.) at the magnification of 200. Five visual fields of each well were randomly chosen.

### Real-time quantitative polymerase chain reaction (RT-qPCR)

Total RNA of CSCs-exo and the tumor cells were extracted by MolPure® Cell RNA Kit (Yeasen Biotechnology Co., Ltd.). Reverse transcription and PCR amplification were carried out using Quant One Step qRT-PCR Kit (Probe) (LM-0102; LMai Biotech Co., Ltd.). All primers were synthesized by Tsingke Biotech Ltd., GAPDH and U6 were used as the internal reference for mRNAs and miRNA, respectively. Fold changes of the RNAs were assessed using 2^−ΔΔCt^ method. The sequences of the primers used were listed in Table 1.

**Table 1:** Primer sequences used for PCR

miR-105-5p	F: 5’-GTGCATCGTGGTCAAATGCT-3’
	R: 5’-ACACCGTAGCACATGCTCAA-3’
GPR12	F: 5’- GGGCTGCCTCGGGATTATTTA-3’
	R: 5’-CACAAGACAATGTCCCAGGGG-3’
ITM2B	F: 5’-TTGCCTCAGTCCTATCTGATTCA-3’
	R: 5’-TCTGCGTTGCAGTTTGTAAGT-3’
ZNF24	F: 5’-CTGATGGCGAAGAGGGATCAA-3’
	R: 5’-CCAGCACTACCAGCTCCAAG-3’
NSL1	F: 5’-CCGCTTCGTGCAAAAGCTC-3’
	R: 5’-TCCAGGATCTTTCTGGGATACTG-3’
GAPDH	F: 5’-ACAACTTTGGTATCGTGGAAGG-3’
	R: 5’-GCCATCACGCCACAGTTTC-3’
U6	F: 5’- GCTTCGGCAGCACATATACTAAAAT-3’
	R: 5’- CGCTTCACGAATTTGCGTGTCAT -3’

### Luciferase reporter assay

The putative binding sequence of wild type (WT) type GPR12 was cloned into the luciferase reporter vectors, while the sequence was mutated and cloned into the vectors as the mutant type (MUT) (synthesized by Shanghai Transheep Biotech Co., Ltd.).The vectors were co-transfected into T24 cells with miR-105-5p mimic/inhibitor using Lipofectamine 2000 (Invitrogen). After culturing for 24 h, the cells were harvested for measuring the luciferase activities using the Firefly Luciferase Assay Kit (ZY130597; Zeye Biotech Co., Ltd.). The activity of *Renilla* was used as an internal control.

### Tumorigenicity assay

BALB/c male nude mice (*n* = 15; six weeks old; weight: 21.2 ± 0.8 g) were purchased from Guangdong Medical Experimental Animal Center. The mice were raised under the SPF condition with a 12 h dark/light cycle. After kept in our lab for a week, xenografts were induced in the mice by subcutaneous injection of 10^7^ T24 cells. After induction for one week, PBS, miR-105-5p antagomir-CSCs-exo or NC antagomir-CSCs-exo were administrated into the mice through intratumor injection (10 μl) thrice a week for total two weeks. Tumor volumes were measured every week following the formula: V = ab^2^/2; a is the long diameter, b is the short diameter. The mice were sacrificed by 150 mg/kg pentobarbital *i.v.* after 30 days of modeling, and the tumors were collected, weighed and embedded for further use.

### Hematoxylin and eosin (H&E) staining

Paraffin sections of the tumor tissues were administrated to H&E staining assay to assess tissue damage degree. The sections were stained with hematoxylin for 5 min and counterstained by eosin for 3 min. Five fields of the stained slide were randomly captured by an optical microscope.

### Statistics

Each experiment was performed for triplication. Data were analyzed by GraphPad Prism (version 9.1.1.225, GraphPad Software Inc.). The data were presented as mean ± SD. Student t-test was performed for the two-group comparison, and analysis of variance (ANOVA) was used for the comparison among multiple groups followed by Duncan’s post-hoc test. *P* < 0.05 was regarded as a significant difference.

## Results

### Isolation of exosomes from CSCs

**Fig. 1:**
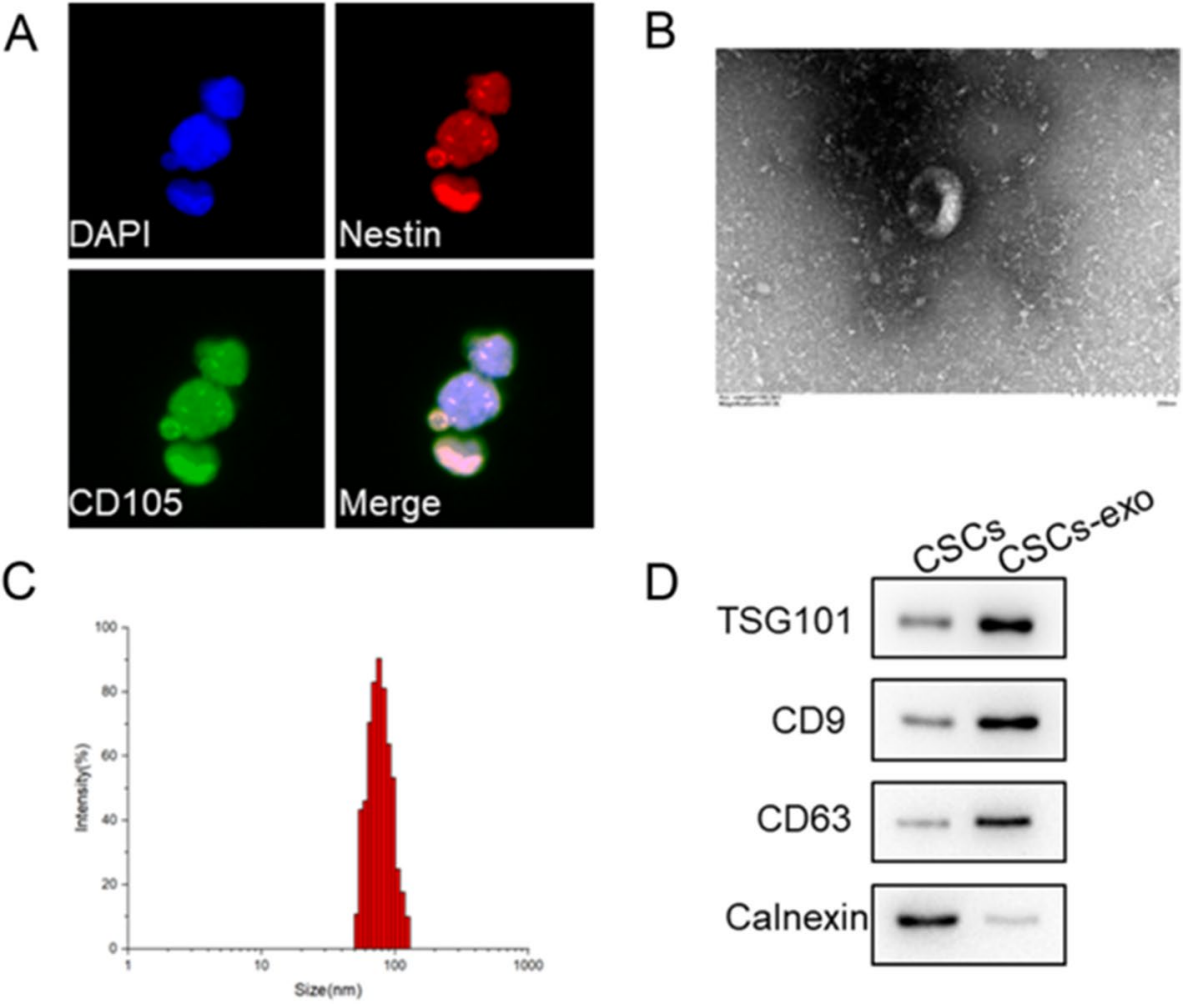
CSCs and CSCs-exo were identified. The displayed results were obtained from T24 cell line. **A** The markers of CSCs, CD105 and Nestin, were examined by immunofluorescence assay. **B** The morphology of CSCs-exo was observed under transmission electron microscope. **C** Sizes of CSCs-exo were analyzed by nanoparticle tracking analysis. **D** The biomarkers of CSCs-exo, TSG101, CD63 and CD9, were examined by western blot assay. Calnexin was used as a negative marker for CSCs-exo. *CSC* Cancer stem cells, *BCSC* Bladder cancer stem cells, *CSCs-exo* Exosomes derived from cancer stem cells

First, CSCs were isolated from the cultured tumor spheres, and the markers of BCSCs were examined. CD44 is an acknowledged surface marker for BCSCs, and CD105 and Nestin are the generally used markers of CSCs. As shown in Fig. 1A, CD105 and Nestin were both positively expressed of the T24 spheres, indicating high stemness of the tumor spheres. Next, CSCs-exo were extracted and authenticated. The isolated vesicle presented as a bowl-shape under TEM observation (Fig. 1B), and diameters of the vesicles were mainly concentrated in 70–110 nm (Fig. 1C). Furthermore, expressions of TSG101, CD63 and CD9 were positive in CSCs-exo, while Calnexin was negative (Fig. 1D). These results proved the nature of CSCs-exo.

### CSCs-exo promoted BC cell viability, migration and invasion

**Fig. 2:**
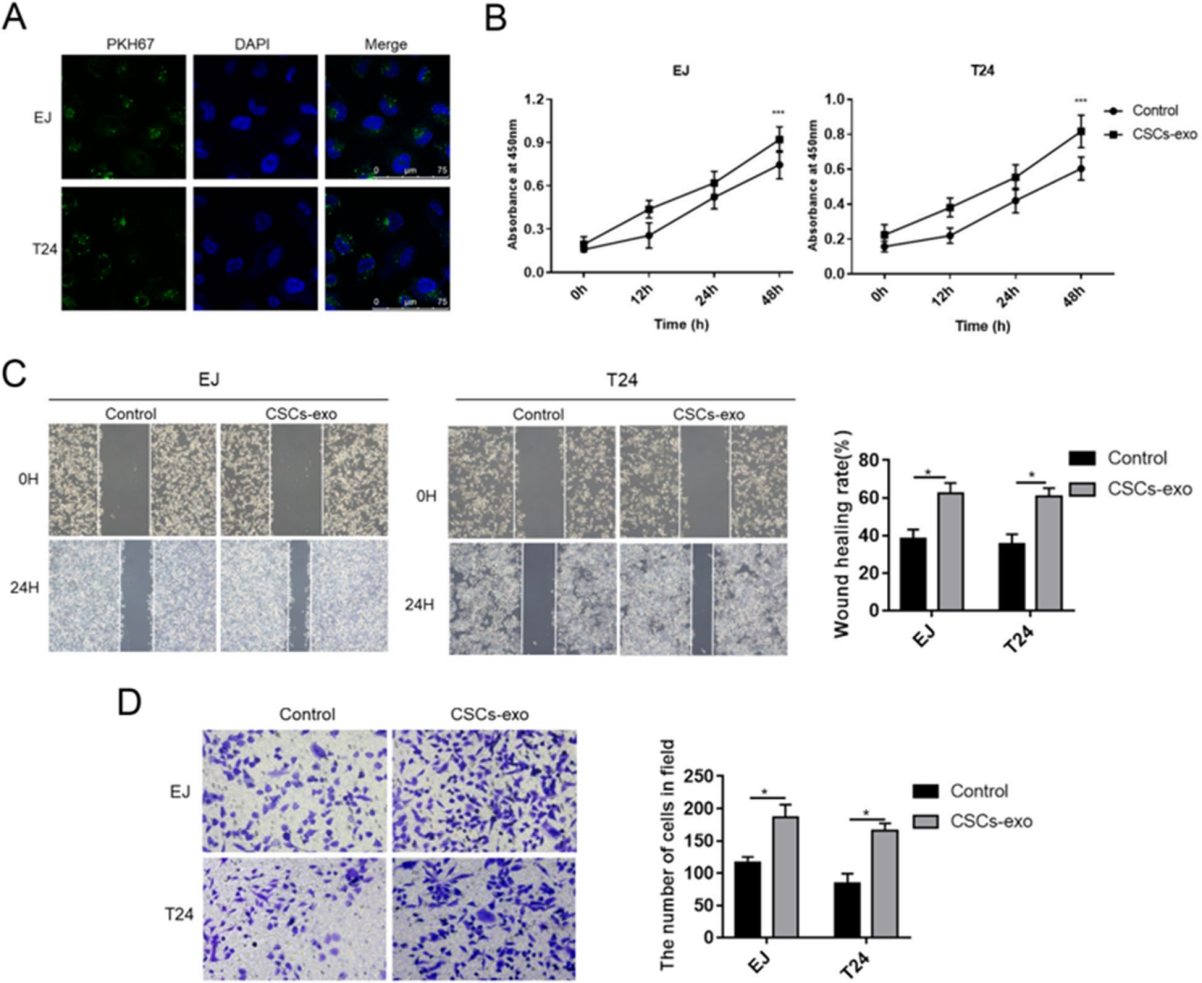
CSCs-exo promoted BC cell viability, migration and invasion. **A** CSCs-exo was stained by PKH67 to assess cellular uptake of EJ and T24 cells. **B** Cell viabilities of EJ and T24 cells were detected by CCK-8 assay. **C** Cell migration abilities of EJ and T24 cells were evaluated by wound healing assay. The wound gap was measured at 0 h and 24 h after wound was scratched. **D** Cell invasion was determined by transwell assay. The invaded cells were counted after 24 h of transwell assay. Experiments were conducted thrice for each approach. ^*^P < 0.05; ^***^P < 0.001. CSCs-exo, exosomes derived from cancer stem cells

The biological functions of CSCs-exo on BC cells were subsequently explored. According to Fig. 2A, PKH67-stianed CSCs-exo could enter into T24 and EJ cells, suggesting the potential of CSCs-exo on regulating BC cells. After CSCs-exo treatment, cell viabilities of T24 and EJ cells were promoted (Fig. 2B). Wound healing rates and invaded cell numbers of EJ and T24 cells were consistently increased by CSCs-exo (Fig. 2C and D).

### miR-105-5p was the effector of CSCs-exo in promoting the aggressiveness of BC cells

**Fig. 3:**
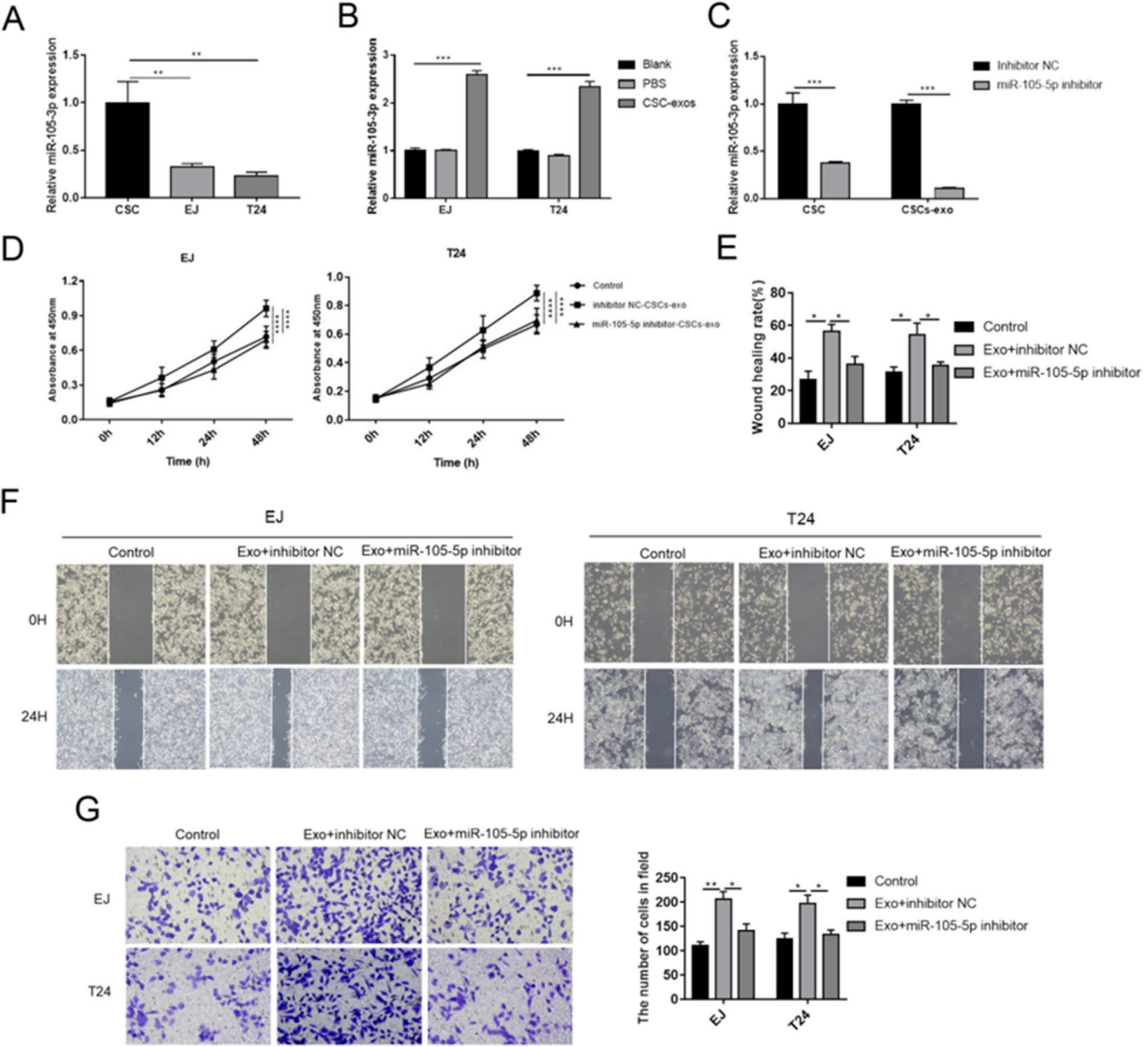
Inhibition of miR-105-5p reversed the carcinogenic effects of CSCs-exo. **A** The expression levels of miR-105-5p in CSCs, T24 and EJ cells were measured by RT-qPCR. **B** The expression levels of miR-105-5p were measured in EJ and T24 cells after incubated with CSCs-exo. **C** miR-105-5p inhibitor was transfected into CSCs, and CSCs-exo. **D** Cell viability, **E** and **F** migration and **G** invasion of EJ and T24 cells were determined after incubation with CSCs-exo. Experiments were conducted thrice for each approach. ^*^P < 0.05; ^**^P < 0.01; ^***^P < 0.001; ^****^P < 0.0001. *CSCs* Cancer stem cells, *CSCs-exo* Exosomes derived from cancer stem cells

Since CSCs-exo was able to exacerbate aggressive behaviors of the BC cells, we further intended to investigate the effector molecule of CSCs-exo. The regulatory effects of exosomes are dependent on the inclusions. miR-105-5p is demonstrated to be upregulated in triple-negative breast cancer [15] and non-small cell cancer [12], but the role of miR-105-5p is still unclear in BC. Hence, the expression of miR-105-5p was detected in CSC, CSCs-exo and BC cells. The expression level of miR-105-5p in CSC was notably higher than EJ and T24 cells (Fig. 3A). In addition, CSCs-exo markedly elevated the level of miR-105-5p in EJ and T24 cells (Fig. 3B). After transfection of miR-105-5p inhibitor, miR-105-5p was significantly downregulated in both CSC and CSCs-exo (Fig. 3C). Inhibition of miR-105-5p in CSCs-exo prominently suppressed the enhancement of cell viabilities in EJ and T24 cells induced by CSCs-exo (Fig. 3D). Similarly, wound healing rates and invaded cell numbers of EJ and T24 cells were decreased by miR-105-5p inhibitor (Fig. 3E-G). These results proved that miR-105-5p was highly expressed in CSCs-exo and participated in regulating cellular behaviors of BC cells.

### GPR12 was the target of miR-105-5p

**Fig. 4:**
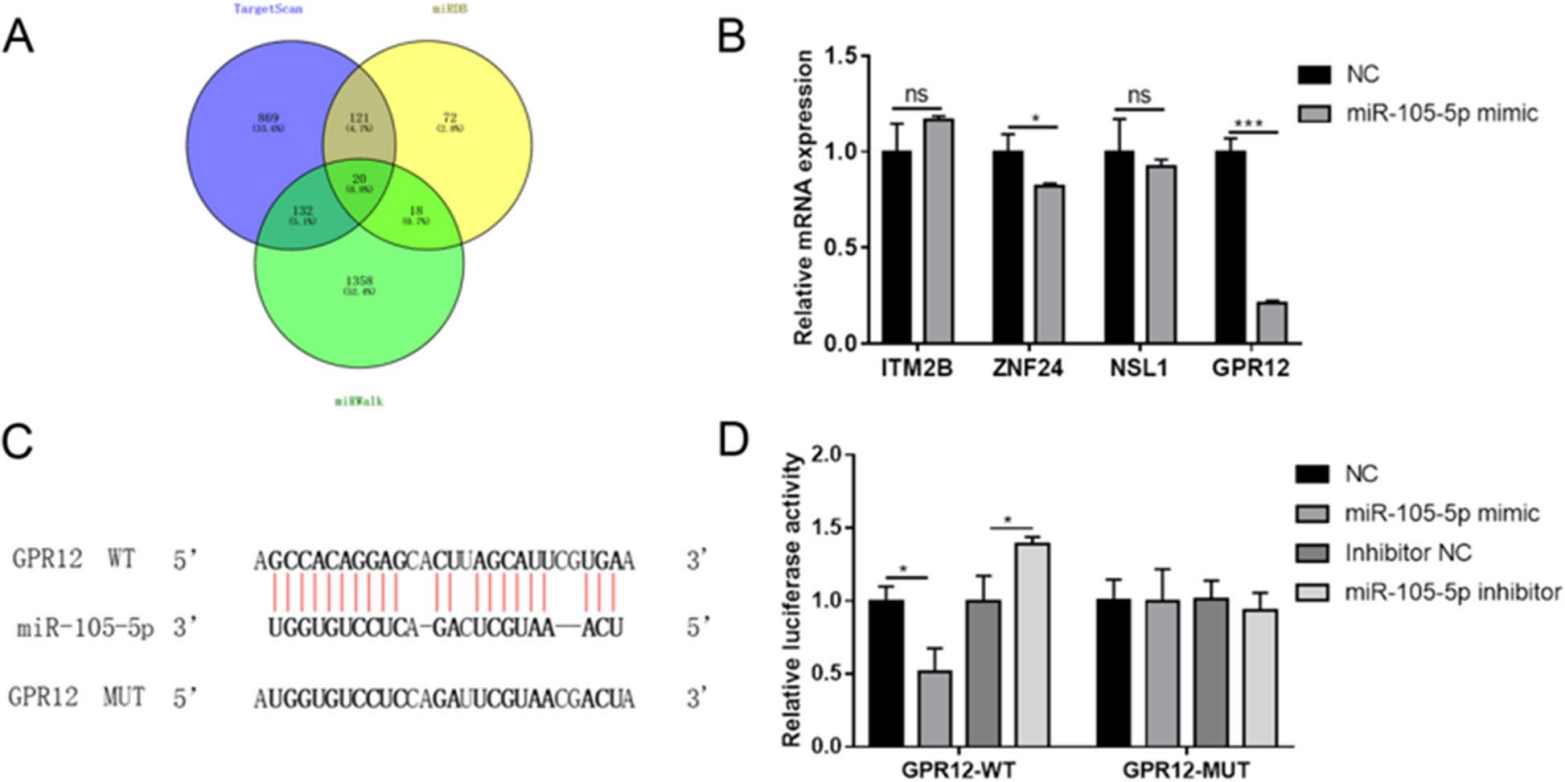
GPR12 was the target of miR-105-5p. **A** The potential targets of miR-105-5p were predicted by miRDB (http://mirdb.org/), miRWalk (http://mirwalk.umm.uni-heidelberg.de/) and TargetScan (http://www.targetscan.org/vert_71/), and the results were expressed as the Venn diagram. **B** mRNA expressions of GPR12, ITM2B, ZNF24, and NSL1 were detected after miR-105-5p mimic was transfected into T24 cells. **C** Sequences of putative binding sites of miR-105-5p and GPR12 were listed, and the mutant binding sequence of GPR12 was designed. **D** Luciferase reporter assay was performed to validate the binding between miR-105-5p and GPR12. Experiments were conducted thrice for each approach. ^*^P < 0.05; ^***^P < 0.001

The regulatory functions of miRNAs are performed through binding to mRNAs, ulteriorly regulating the translation process of the target genes [16]. The putative targets of miR-105-5p were predicted by miRDB, miRWalk and TargetScan. As the Venn diagram displayed, 20 genes were obtained in the intersection part (Fig. 4A). Based on the literatures included in PubMed, GPR12, ITM2B, ZNF24, and NSL1 were selected as the candidate targets due to the low expressions in various cancers [17–20]. We examined the mRNA expressions of these four genes in T24 cells, and GPR12 showed the most significant downregulation after miR-105-5p mimic transfection (Fig. 4B). Subsequently, the WT and MUT sequences of GPR12 were cloned into luciferase reporter genes (Fig. 4C). In the WT group, miR-105-5p mimic remarkably reduced luciferase activity, while miR-105-5p inhibitor increased it. However, luciferase activity of the MUT group was not significantly influenced by miR-105-5p mimic or inhibitor (Fig. 4D).

### GPR12 partially abrogated the carcinogenic role of miR-105-5p

**Fig. 5:**
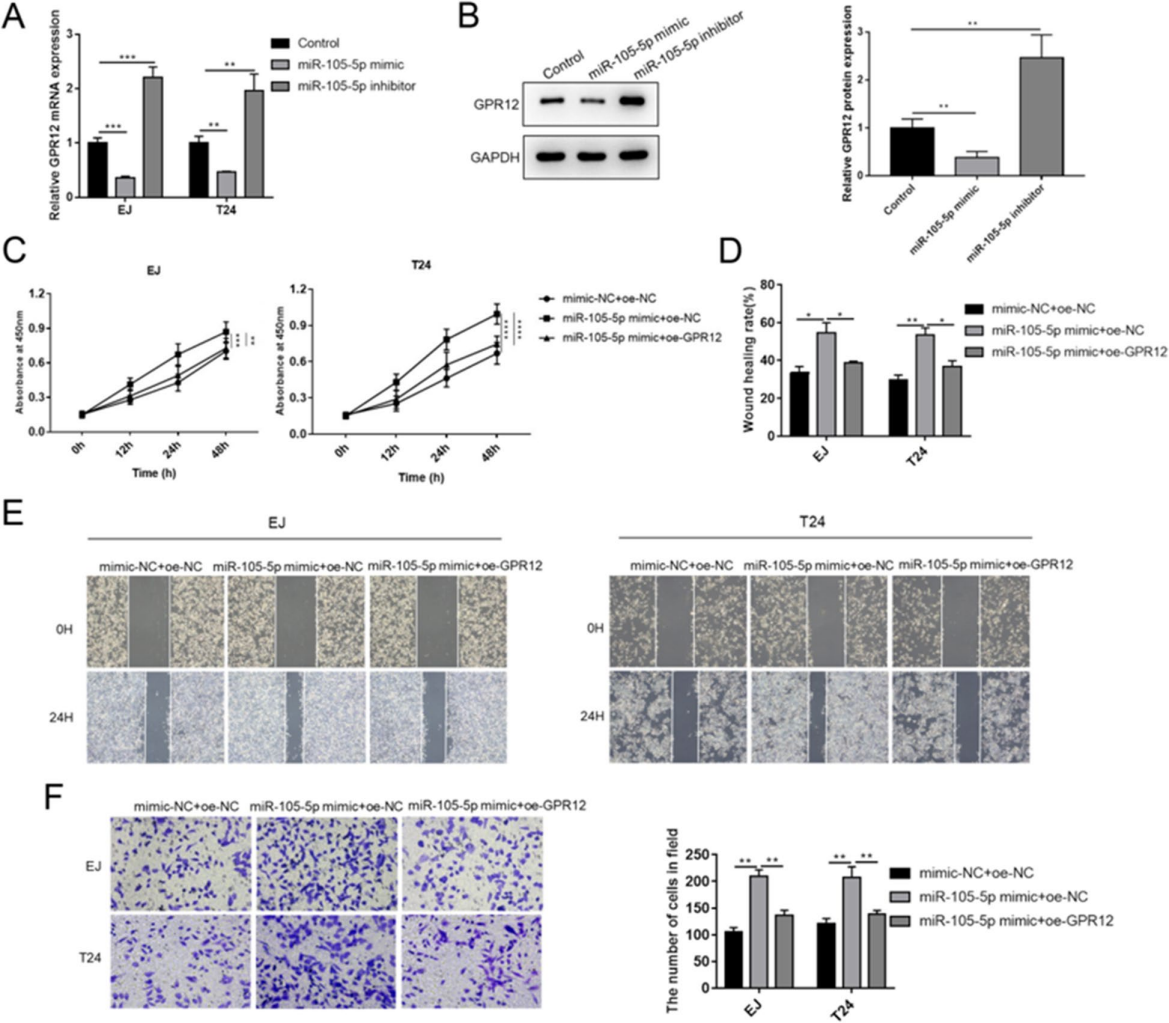
GPR12 overexpression reversed the effects of miR-105-5p on BC cell growth and metastasis. **A** mRNA and **B** protein levels of GPR12 were measured under the treatment of miR-105-5p mimic or inhibitor. **C** Cell viability, **D** and **E** migration and **F** invasion of EJ and T24 cells were determined after transfection with miR-105-5p or GPR12. Experiments were conducted thrice for each approach. ^*^P < 0.05; ^**^P < 0.01; ^***^P < 0.001; ^****^P < 0.0001. BC, bladder cancer

The expression of GPR12 mRNA was significantly inhibited by miR-105-5p mimic, while enhanced by miR-105-5p inhibitor (Fig. 5A). Likewise, the protein level of GPR12 in T24 cells exhibited the similar trend with mRNA level of GPR12 under the treatment of miR-105-5p mimic/inhibitor (Fig. 5B). Transfection of miR-105-5p mimic observably facilitated cell viabilities of EJ and T24 cells; however, GPR12 overexpression reversed the effects of miR-105-5p (Fig. 5C). Wound healing rates of EJ and T24 cells transfected with miR-105-5p mimic showed prominent enhancements, which was abrogated by GPR12 overexpression (Fig. 5D and E). Moreover, miR-105-5p mimic induced notable increments on invaded cell numbers of EJ and T24 cells, but the increments were markedly suppressed after GPR12 overexpression (Fig. 5F).

### Inhibition of miR-105-5p suppressed tumor formation in the nude mice

**Fig. 6:**
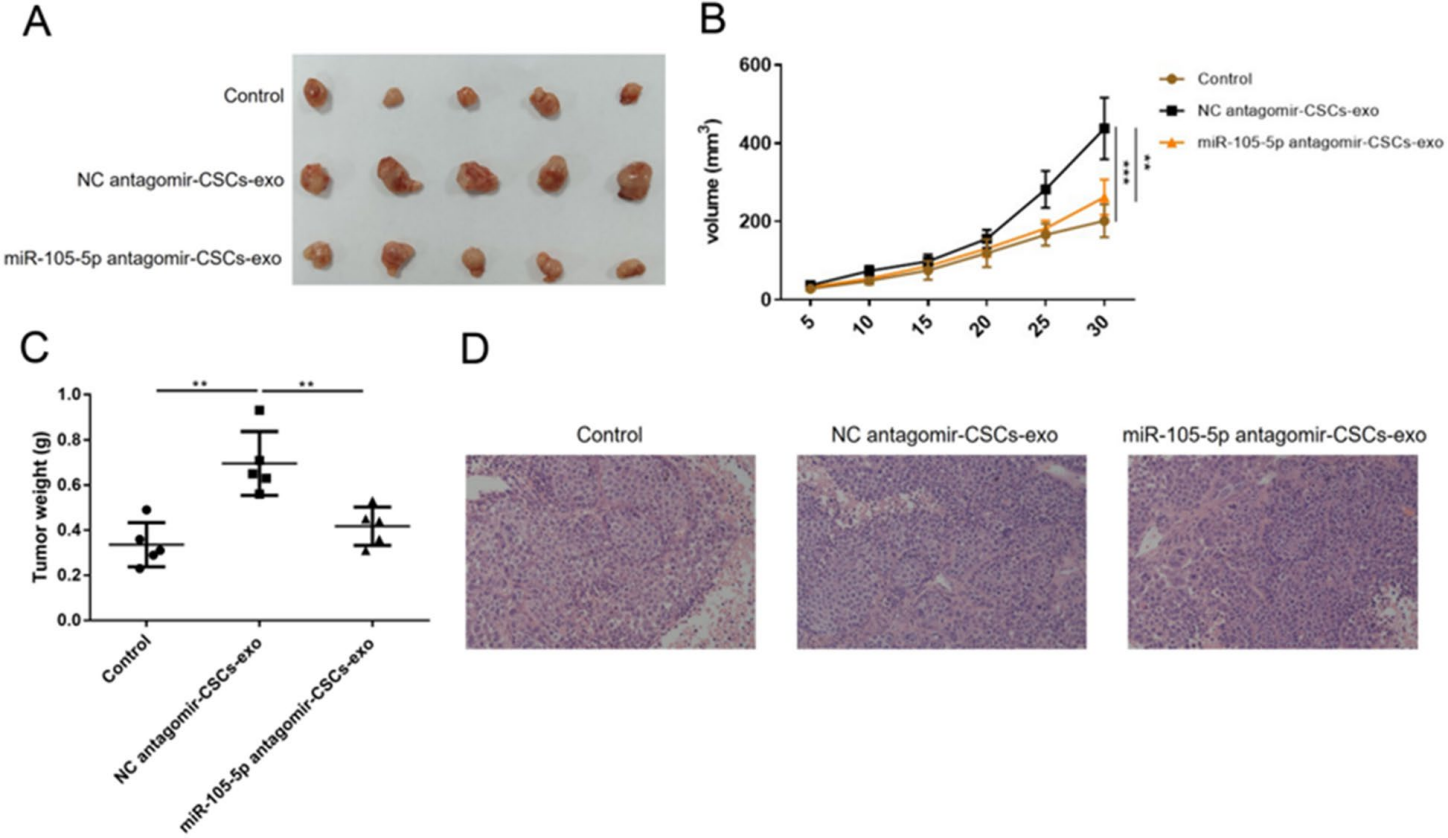
miR-105-5p antagomir suppressed tumor growth in vivo. Tumors were induced in the nude mice through injecting T24 cells. **A** The tumors were resected and photographed after 30 d of induction. **B** Tumor volumes were documented every five days since T24 cells were injected. **C** Tumor weights were measured after resection. **D** Histological changes of tumors were assessed by H&E staining. ^**^P < 0.01; ^***^P < 0.001. *BC* Bladder cancer

Finally, xenografts were induced in the nude mice to examine the role of miR-105-5p in vivo. The tumors were resected as displayed in Fig. 6A. CSCs-exo accelerated tumor growth, and miR-105-5p antagomir impaired the tumorigenic function of CSCs-exo (Fig. 6B). Additionally, the increasement of tumor weights triggered by CSCs-exo was decreased by miR-105-5p antagomir (Fig. 6C). H&E staining images showed that CSCs-exo conferred higher malignancy to the tumors, but miR-105-5p antagomir partially relieved the malignancy (Fig. 6D).

## Discussion

BCSCs are a population of tumor microenvironment (TME) that are closely related to tumor behavior, and exosomes are the main transmitters circulated in TME that are responsible for intercellular communication between BCSCs and other cells [11]. In this study, we identified exosomes from BCSCs, and proved that the exosomes could promote cell growth and metastasis of BC cells. Based on the previous researches, miR-105-5p is defined as a functional miRNA and shows an oncogenic role in triple-negative breast cancer [15] and non-small cell cancer [12]. We demonstrated that miR-105-5p was the effector of CSCs-exo on aggravating BC, and GPR12 was elucidated to be its target gene.

CSCs are a small subset of stem cell-like tumor cells that exist in tumor tissues with unlimited self-renewal ability and play a decisive role in initiating tumor formation and growth. Thus, boosting the development of CSCs-related researches on carcinogenic mechanism is of great importance to oncology. Tumor cells with high Nestin and CD105 levels are acknowledged as CSCs [21, 22]. In the current study, we cultured T24 cells in the specific serum-free medium to induce sphere formation of the cells. The propagated sphere cells showed positive expressions of CD105 and Nestin, manifesting the hallmark of self-renewal. Exosomes are essential mediators in intercellular communication. For example, colorectal CSC-derived exosomal RNAs induced the expression of IL-1β through a pattern recognition-NF-κB signaling axis to sustain neutrophil survival and elevate tumor formation [23]. Exosomes from glioma stem cells (GSCs-exo) significantly promoted proliferation and invasion of glioma cells due to the highly enriched Notch1 protein in GSCs-exo [24]. Yang et al. [25] found that gemcitabine (GEM)-resistant pancreatic cancer stem cells could enhance the drug resistance of GEM-sensitive pancreatic cancer cells by transmitting exosomal miR-210. In this paper, the promotive effect of CSCs-exo on cellular properties of BC was investigated.

Further, the relationship between miRNAs and CSCs-exo in BC was probed. Upregulation of miR-944 in GSCs led to miR-944 enrichment in the GSCs-exo, thus markedly decreasing proliferation, migration, and tube formation of human umbilical vein endothelial cells to suppress glioma growth [26]. Lung CSC-derived exosomal miR-210-3p promoted EMT process of lung cancer cells via binding to 3’-UTR of FGFRL1 [27]. As for BCSCs, downregulation of miR-200c was correlated with the inhibited stemness [28]. Isorhapontigenin-induced miR-4295 could bind to USP28 mRNA and inhibit its translation and expression, so as to attenuate stemness and invasivity of BCSCs [29]. Nevertheless, the previous studies mainly focus on the regulation of miRNA on the properties of CSCs, but the effects of CSCs-derived miRNAs on tumor cells are rarely investigated. As a type of post-transcriptional regulator, miRNAs have been reported to take part in malignant behaviors such as tumor proliferation, invasion and metastasis [30]. miR-105-5p was proved to be downregulated in glioma [31] and cervical cancer [32], but overexpressed in non-small cell cancer [12] and triple-negative breast cancer [15] as early diagnostic biomarkers. In liver cancer, low level of miR-105-5p induced higher stemness of CSCs and PES1 accumulation [33]. In our study, CSCs-exo was observed to notably increase the miR-105-5p levels in T24 and EJ cells, thereby facilitating tumor cell viability, migration and invasion. Transfection of miR-105 inhibitor into CSCs mitigated the tumorigenic function of CSCs-exo. Our results first emphasized the oncogenic function of miR-105-5p on BC cells that was transmitted by exosomes from CSCs.

The molecular mechanism of miR-105-5p was further explored, and GPR12 was proved to be the target of miR-105-5p. GRP12 is a member of G protein-coupled receptors that is capable of sending signals through G protein-mediated or non-G protein-mediated mechanisms [34]. Overexpression of GPR12 induced keratin 8 phosphorylation and reorganization, which might contribute to cancer cell metastasis [35]. Reversely, GPR12 was restrained in the esophageal cancer (EC) and hypopharyngeal cancer (HC) tissues; restored expression of GPR12 in EC and HC promoted tumor cell apoptosis by activating caspase-7 [17]. In the current research, we elucidated that the mRNA and protein expression levels of GPR12 were negatively correlated with miR-105-5p, and overexpression of GPR12 abrogated the oncogenic effects of miR-105-5p. Collectively, GPR12 might serve as a tumor suppressor in BC, but the exploration of detailed mechanism was in need.

## Conclusions

To sum up, the present results demonstrated that BCSCs conferred malignancy to BC cells by transmitting exosomal miR-105-5p, and GPR12 was the target of miR-105-5p. These findings provided a promising diagnostic marker for BC and therapeutic targets for CSC targeted therapies.

### Supplementary Information

**Additional file 1: Figure S1. **Full length gels and blots of proteins. Full length gels and blots of TSG101 (the first row and first column), CD9 (the first row and second column), CD63 (the first row and third column), Calnexin (the second row and first column), GPR12 (the second row and second column) and GAPDH (the second row and third column) were shown in order.

## Data Availability

The datasets used and/or analyzed during the current study are available from the corresponding author on reasonable request.
