# Supplementary figures and images for: Exosomal miR-105-5p derived from bladder cancer stem cells targets for GPR12 to promote the malignancy of bladder cancer
Source: BMC Urol. 2023 Oct 3;23:155. doi: 10.1186/s12894-023-01326-2 (PMC10548737; doi:10.1186/s12894-023-01326-2)

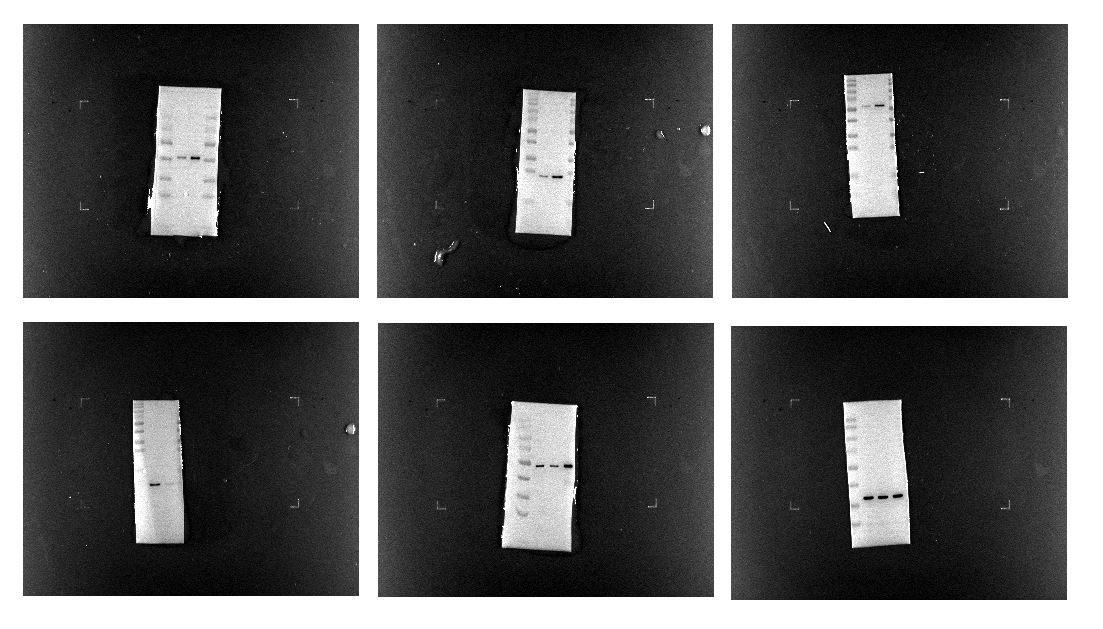

Supplement: Supplementary file 1 — Additional file 1: Figure S1. Full length gels and blots of proteins. Full length gels and blots of TSG101 (the first row and first column), CD9 (the first row and second column), CD63 (the first row and third column), Calnexin (the second row and first column), GPR12 (the second row and second column) and GAPDH (the second row and third column) were shown in order. [file 12894_2023_1326_MOESM1_ESM.tif]
